# Development of a Micromobility Riding Evaluation Platform for an Indoor Riding Lane Based on Multi-View Overhead Video Integration

**DOI:** 10.3390/s26175614

**Published:** 2026-09-03

**Authors:** Kimihiko Iwata, Makoto Shinnishi, Takashi Hikasa, Mutsumi Suganuma, Satoshi Takahashi

**Affiliations:** 1 Nagoya Electric Works Co., Ltd., Ama 490-1294, Aichi, Japan; stakahashi@nagoya-denki.co.jp; 2 School of Management and Information Sciences, Tama University, Tama 206-0022, Tokyo, Japan; shinnishi-m@tama.ac.jp (M.S.); hikasa@tama.ac.jp (T.H.); suganuma-m@tama.ac.jp (M.S.)

**Keywords:** multi-camera overhead video, micromobility, e-scooter, deep learning-based object detection, evaluation platform

## Abstract

This study developed a simple and scalable e-scooter riding evaluation platform that reduces on-site implementation effort by relying primarily on image analysis. The platform supports repeated riding trials under consistent conditions in an indoor environment. By combining the areas covered by two drones, the system recorded an entire long and narrow indoor riding lane. A pretrained YOLO model was fine-tuned to construct a rider detection model adapted to the experimental environment. ORB-based image registration and trajectory integration then transformed the riding trajectories obtained from the two cameras into a common coordinate system. Riding speed, riding duration, and the radius of curvature of the two curves were calculated. The results revealed differences among subjects in speed variation, stability across riding trials, and turning characteristics, including stable low-speed riding, sustained high-speed riding, and deceleration before turning. For most subjects, the inter-camera junction discrepancy was within 10 cm, indicating general internal consistency of trajectory integration under the experimental conditions. These results suggest that the proposed system can serve as a video-based platform for quantitatively evaluating observable riding behavior, including riding trajectory and speed characteristics, in an indoor riding lane.

## 1. Introduction

In recent years, micromobility, including electric scooters (e-scooters), has rapidly emerged as a new mode of urban transportation. These vehicles have the potential to reduce the environmental impact of last-mile travel and improve transportation efficiency. Therefore, they are becoming an important component of urban transportation systems. However, their increasing use has also raised safety concerns, including single-vehicle falls and collisions with pedestrians [1,2].

Previous studies on e-scooter safety have mainly examined crash occurrence, injury characteristics, and relationships with the urban environment. Kazemzadeh et al. reviewed studies in the transportation and medical fields and highlighted the importance of protective equipment and operational regulations [1]. Yang et al. analyzed crash reports and identified relationships with user attributes, crash types, locations, facility conditions, time of occurrence, and injury severity [2]. Bai et al. analyzed the relationship between shared e-scooter use and the urban built environment [3]. A recent naturalistic riding study also showed that riding experience and behaviors such as one-handed riding, mobile phone use, and group riding affect crash risk [4]. These findings indicate that micromobility safety assessments should consider not only crash frequency and injury characteristics but also riding behavior, riding skills, and differences among subjects.

Methods for measuring micromobility riding behavior can be broadly classified into onboard and non-contact sensing. Onboard sensing involves attaching measurement devices to the vehicle or rider. In contrast, non-contact sensing externally observes the rider and vehicle using cameras, LiDAR, or other devices.

Onboard sensing can acquire information on position, speed, acceleration, posture, and the surrounding environment at a high temporal resolution. Prabu et al. developed a measurement platform that combined wearable and onboard sensors to collect naturalistic e-scooter riding data [5]. Naturalistic cycling data have also been used to identify factors that affect bicycle safety in real-world environments [6]. Pérez-Zuriaga et al. developed a low-cost e-scooter data acquisition system capable of measuring position, speed, acceleration, vibration, and lateral clearance [7]. Although these methods provide detailed motion data, sensors must be installed on each vehicle or rider. In addition, the measurement configuration may depend on the vehicle type and experimental conditions, increasing the effort required for experimental preparation and operation. The attached sensors may also affect riding behavior and constrain comparisons among multiple subjects under identical measurement conditions.

Non-contact sensing enables riding behavior to be observed externally without attaching sensors to the rider or vehicle. Optical motion capture is a representative example and enables highly accurate motion measurement. However, calibrated cameras must be arranged around the measurement area [8,9]. Applying this approach to a long riding lane therefore requires expensive equipment and imposes substantial constraints on the experimental layout. Three-dimensional LiDAR has also been used for object detection and tracking, but equipment cost and occlusion remain challenges [10]. Ground-installed cameras can measure a wide area. However, many cameras are required to avoid occlusion while maintaining sufficient image resolution [11].

Computer vision has also been applied to the analysis of bicycle and micromobility riding behavior. Using videos acquired on public roads, Neshagaran et al. analyzed the choice of riding space, helmet use, and relationships between user attributes and riding behavior among e-scooter riders and cyclists [12]. De Bock and Verstockt extracted riding lines, mounted and dismounted states, and segment traversal times from short analysis regions defined in fixed-camera videos of cyclocross races [13]. Verstraete et al. detected riders in overhead videos of the finish section of bicycle races. They estimated homographies between overlapping images to generate spatiotemporal riding-line maps [14]. These studies demonstrated that riding positions, riding lines, and specific user behaviors can be obtained from videos without contact. However, they mainly examined group-level observations on public roads, competitive riding, or short local segments. A framework for comprehensively evaluating speed variations, stability across repeated riding trials, and turning characteristics of the same subject over an entire long and narrow riding lane has not been sufficiently investigated.

Studies have also evaluated the dynamic performance and riding characteristics of personal mobility vehicles under controlled conditions. Paudel et al. used a mathematical model to analyze the effects of rider posture on e-scooter stability, maneuverability, and braking performance [15]. Their study clarified the relationship between e-scooter characteristics and rider posture. However, it did not evaluate differences among subjects or riding trials using measured riding trajectories. Nishiuchi et al. acquired Segway riding videos using a single fixed camera installed on the sixth floor of a building adjacent to a test course [16]. Image coordinates were transformed into riding trajectories on the course plane using projective transformation. The authors then compared the riding behavior of novice and experienced riders during acceleration, deceleration, slalom riding, overtaking, passing pedestrians, and emergency braking.

Camera-based analysis systems such as that of Nishiuchi et al. do not require numerous sensors to be attached to riders or vehicles. They can therefore reduce the effort required for on-site deployment and operation. However, acquiring comparable overhead videos requires a building or other elevated structure near the experimental site on which the camera can be installed. The available recording area is also restricted because the camera position and viewing angle depend on the location and structure of the building. Consequently, such systems have limited flexibility in modifying the course layout, placing obstacles in the experimental field, and extending the measurement section.

Drone-based trajectory measurement has been used to reduce the installation constraints of fixed cameras and capture wide areas from overhead viewpoints. Representative examples include the highD dataset for highways [17], the inD dataset for intersections [18], and the pNEUMA dataset for urban traffic [19]. These large-scale datasets contain detailed naturalistic trajectories of vehicles, pedestrians, and other road users and are useful for microscopic traffic-flow analysis. Drones also enable overhead viewpoints to be selected according to the measurement target without installing cameras on surrounding buildings.

However, highD, inD, and pNEUMA were acquired in outdoor, real-world traffic environments. Weather, lighting, traffic demand, interactions among road users, and road-surface conditions therefore cannot be fully controlled. Furthermore, these datasets were not designed to repeatedly expose the same subject to the same riding task or to compare changes between riding conditions or before and after training. A measurement platform is therefore needed that combines the flexibility of drone-based overhead recording with repeated riding trials in a controlled indoor environment.

Multi-camera object detection and tracking methods have also been developed to reduce occlusion and missed detections over wide measurement areas. WILDTRACK provides synchronized videos from seven calibrated cameras installed in a public space and is used to evaluate multi-view pedestrian detection and trajectory estimation [20]. MVDet improves detection performance under occlusion by projecting feature maps from multiple cameras onto a common ground-plane coordinate system [21]. Methods that integrate multi-camera detection and tracking results into a bird’s-eye-view coordinate system have also been proposed for pedestrians and vehicles [22]. These studies show that integrating multiple viewpoints can reduce the limitations of occlusion and field of view associated with a single camera. However, their primary objective is object detection and tracking in crowded or general traffic environments. They do not primarily address the continuous integration of e-scooter riding trajectories from multiple cameras or the evaluation of riding characteristics from repeated trials by the same subject in a controlled indoor environment.

Image registration and object detection are key technologies for video-based trajectory analysis. Szeliski identified feature correspondence, geometric transformation estimation, and blending as the principal components of image alignment and image stitching [23]. ORB enables computationally efficient feature extraction [24], whereas RANSAC is widely used for robust geometric transformation estimation in the presence of outliers [25]. These techniques have been applied to drone image registration [26,27], and methods based on deep features have also been investigated [28]. YOLO-based models enable high-speed object detection [29]. By fine-tuning a model pretrained on a general-purpose dataset with images acquired in the target experimental environment, an environment-specific object detection model can be developed from a relatively small custom dataset rather than trained from scratch. This approach reduces the effort required to create a training dataset and train the model while enabling adaptation to the target and experimental environment. DeepSORT and ByteTrack associate detected objects across frames and have contributed to improved multi-object tracking performance [30,31]. However, applying these individual technologies to repeated riding evaluation in a long and narrow indoor riding lane requires their integration according to the lane geometry, camera arrangement, and riding metrics of interest.

The existing methods therefore have different advantages and limitations. Onboard sensing provides detailed motion information but requires devices to be attached to the vehicle or rider. Optical motion capture provides high measurement accuracy, but its application to a long riding lane is constrained by cost and camera arrangement. Fixed-camera video analysis enables non-contact measurement, but the available camera positions depend on the surrounding environment. This dependence restricts the measurement range and modification of the course layout. Outdoor drone-based trajectory measurement offers flexible camera positioning but is unsuitable for repeated trials under controlled experimental conditions. In addition, existing multi-camera studies have primarily focused on general object detection and tracking.

Evaluating e-scooter safety, riding skills, training effects, and responses to road signs and obstacles requires an environment in which the same riding tasks can be repeated under comparable conditions. Indoor environments can reduce variations in weather, lighting, and surface conditions. A practical evaluation system should not require densely arranged and expensive motion-capture equipment or onboard sensors. It should also allow the camera positions and measurement range to be adjusted according to the course layout. Furthermore, the system should continuously acquire riding trajectories over an entire long and narrow riding lane and enable quantitative comparisons among subjects.

Accordingly, this study proposes a non-contact and non-invasive platform for acquiring and analyzing e-scooter riding trajectories in a long and narrow indoor riding lane. The hardware configuration was simplified as much as possible to reduce the effort required for on-site deployment and operation. The required measurement functions were supplemented by image analysis. Cameras mounted on two drones hovered at fixed positions above the riding lane and acquired vertically downward overhead videos. The use of drones eliminated the need to install cameras on surrounding buildings and allowed the recording positions to be adjusted according to the course layout. The recording ranges of multiple drones were combined to extend the measurement area along a long riding lane that could not be recorded by a single camera while maintaining sufficient image resolution.

To detect rider positions in the acquired videos, a YOLO model pretrained on a general-purpose dataset was fine-tuned using custom images acquired in the experimental environment. Compared with training a model from scratch, this approach enabled the development of an upper-body detection model adapted to the recording direction and appearance of the riders using a relatively small training dataset. ORB-based image registration and trajectory integration were then used to transform the riding trajectories obtained from each camera into a common coordinate system. Riding speed, riding duration, and the radius of curvature of each curve were subsequently calculated to evaluate differences in riding characteristics among subjects.

The main contributions of this study are as follows. (1) A simple and scalable drone-based multi-camera trajectory measurement system was developed for repeated e-scooter riding trials in a long and narrow indoor riding lane. (2) Rider detection based on fine-tuning a pretrained YOLO model was combined with ORB-based image registration and trajectory integration. This combination enabled riding trajectories to be obtained over the entire long and narrow measurement area using a relatively small custom training dataset. (3) The platform enabled riding trajectories, riding speed, riding duration, and turning characteristics to be compared among subjects under controlled experimental conditions without requiring them to wear sensors. This platform is not intended to replace outdoor naturalistic riding observations. Instead, it provides a complementary approach for evaluating micromobility riding behavior under reproducible conditions.

## 2. Materials and Methods

### 2.1. Proposed System

**Figure 1:**
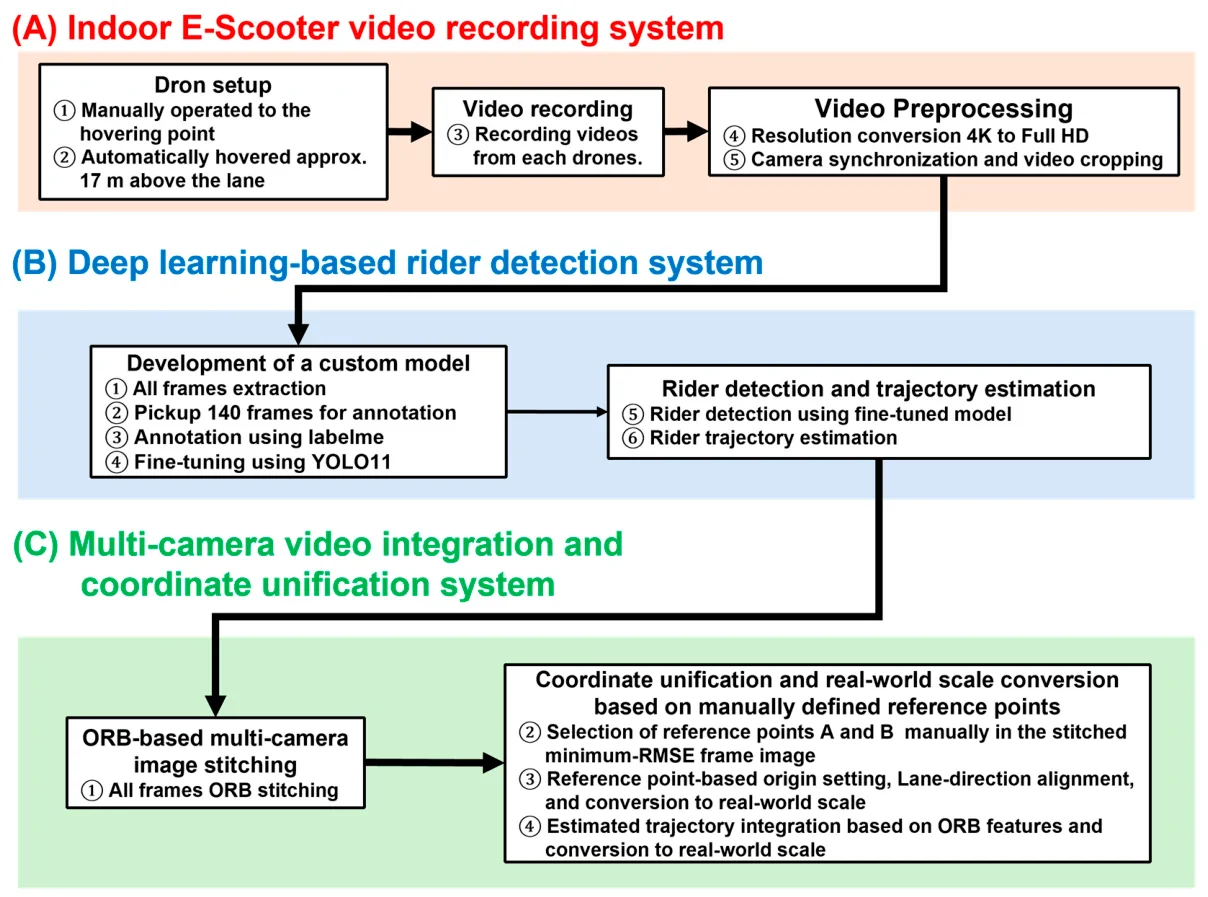
Overall system configuration.

In this study, we developed a riding trajectory analysis system using videos captured by multiple cameras. The system was designed to reduce the burden of on-site data collection and make it easier to conduct experiments at many different locations. It enables the quantitative evaluation of e-scooter riding behavior on an indoor riding lane. An overview of the system is shown in Figure 1. The system consists of three components: (A) an indoor e-scooter video recording system, (B) a deep learning-based rider detection and riding trajectory estimation system, and (C) an ORB-based image registration and trajectory integration system.

In the indoor e-scooter video recording system, two drones hover above the riding lane and record the rider from overhead. The recorded videos are resized and then divided into separate videos for individual riding trials.

In the deep learning-based rider detection and riding trajectory estimation system, YOLO11 is fine-tuned using riding images extracted from the recorded videos. The fine-tuned model is used as a custom rider detection model. Rider detection is performed on every frame of each riding trial. The riding trajectory is then estimated from the detected rider positions.

In the ORB-based image registration and trajectory integration system, image registration parameters that minimize the root mean square error (RMSE) are estimated for each riding trial. The frame images are then stitched based on ORB feature matching. In addition, the coordinate systems are standardized across riding trials using known reference points and a known reference distance in the videos. Finally, the image coordinates are converted into real-world coordinates.

### 2.2. Indoor Riding-Lane Recording System Using Two Drones

**Figure 2:**
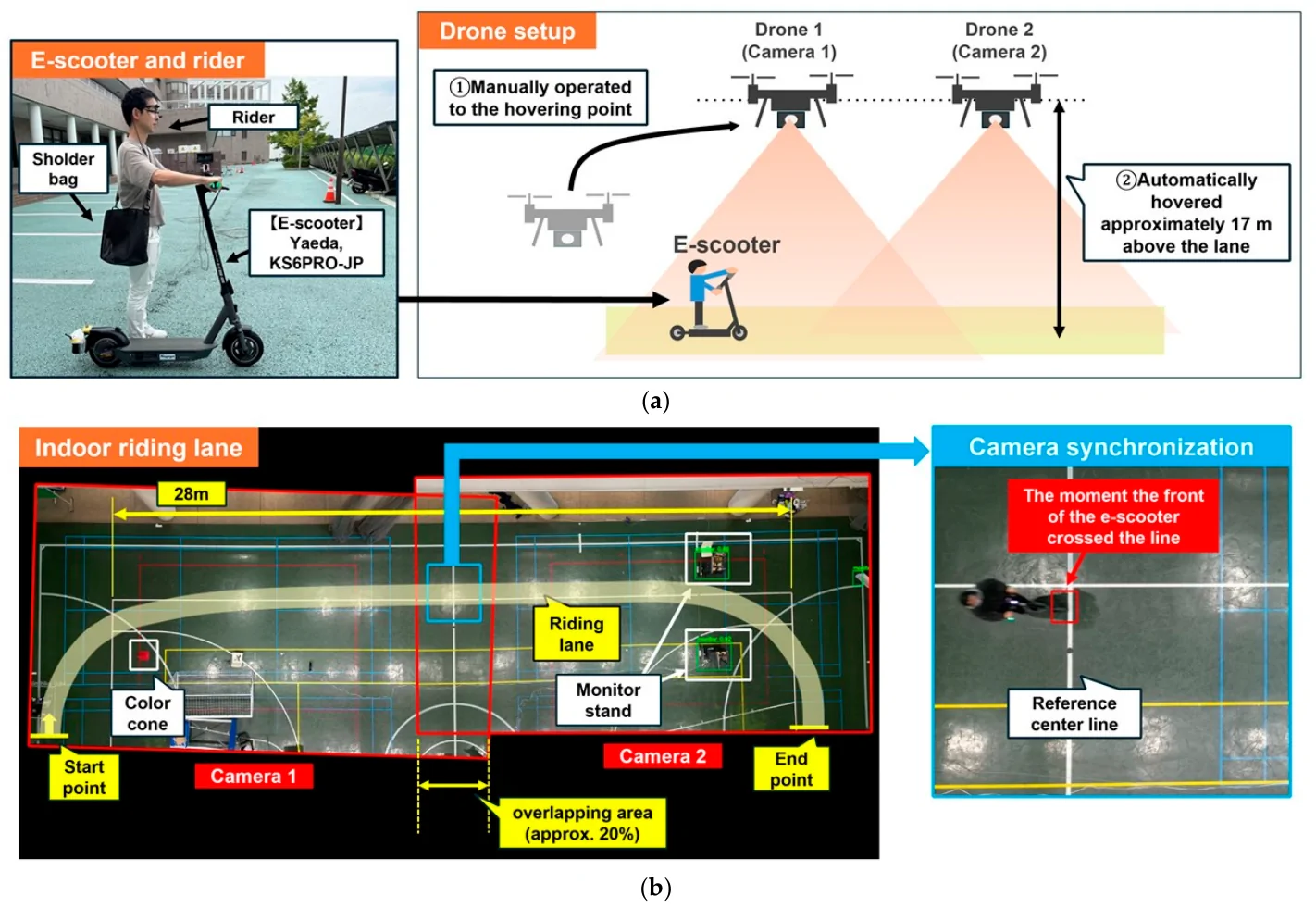
(**a**) Transportation to the drone recording site. (**b**) Drone imagery and riding lane setup.

A video recording system using two drones was developed to capture the riding behavior of e-scooter riders on a long, narrow indoor riding lane. The riding lane was divided into first and second sections. Each section was recorded from above by a separate drone. The e-scooter and drone arrangement is shown in Figure 2a.

A YADEA KS6PRO-JP e-scooter (Yadea Technology Group Co., Ltd., Wuxi, China) was used in the experiment. Each rider carried a shoulder bag while riding along the lane to represent everyday riding conditions. Two DJI Mini 3 Pro drones (SZ DJI Technology Co., Ltd., Shenzhen, China) were used for video recording. Their camera specifications are listed in Table 1.

The drones were manually positioned approximately 17 m above the first and second sections of the riding lane. This altitude was close to the maximum available height in the gymnasium. The drones were then maintained in flight using their automatic hovering function. Their positions were manually adjusted by visually checking the live video feeds displayed on the controllers. As shown in Figure 2b, Camera 1 and Camera 2 were positioned so that their fields of view overlapped by approximately 20%. The videos were recorded at 4K resolution and 60 fps.

At the starting point, a staff member signaled that the rider could begin. The rider then started the riding trial at a self-selected time.

**Figure 3:**
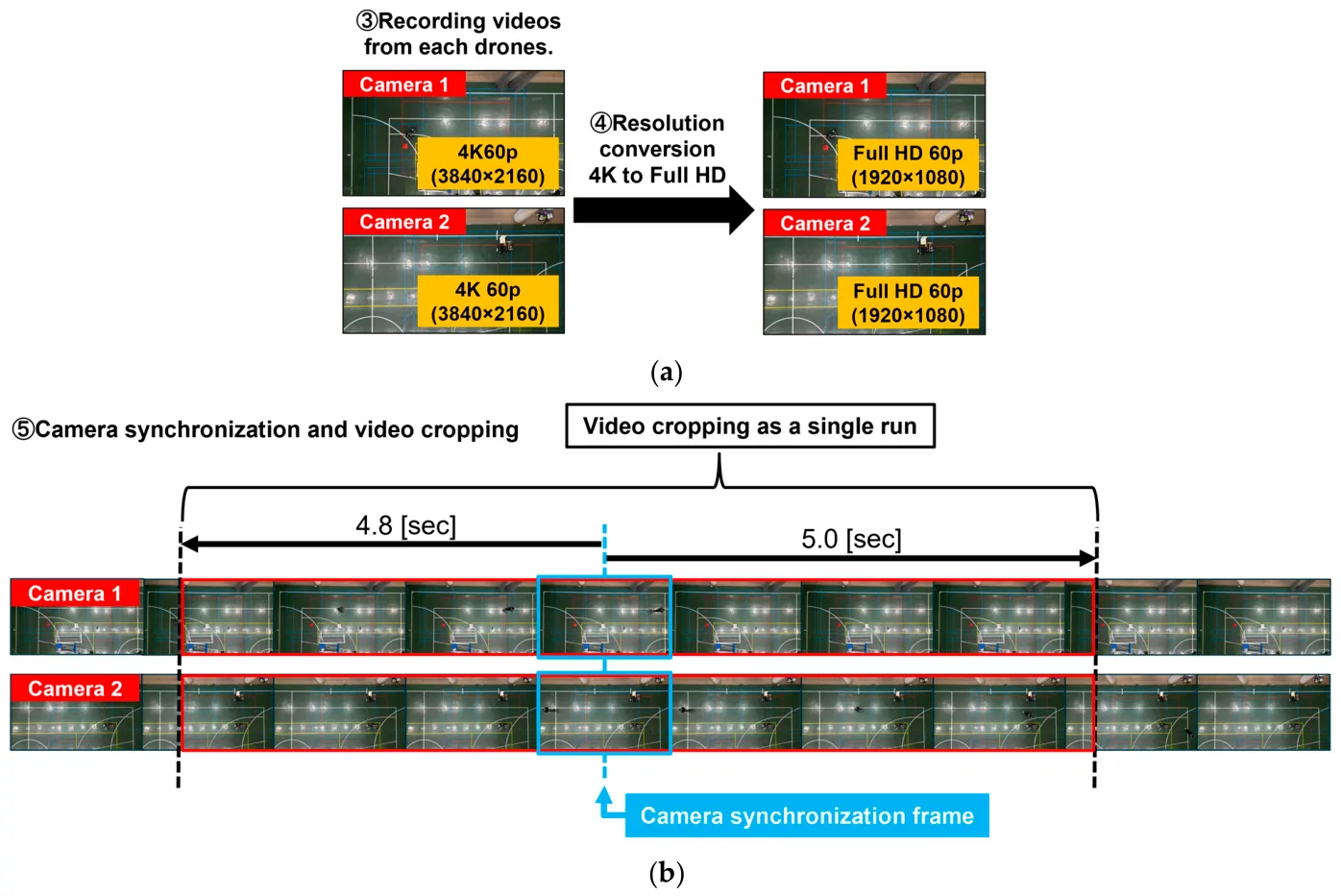
(**a**) Drone video recording and resizing for analysis. (**b**) Camera synchronization and video cropping for each riding trial.

After all riders had completed all riding trials, the riding behavior was analyzed offline. As shown in Figure 3, the recorded videos were converted to 1080p at 60 fps to reduce computational cost and ensure stable processing. The time at which the front end of the e-scooter crossed the white line in the overlapping region was used as the synchronization reference for the two cameras. For each riding trial, both videos were cropped from 4.8 s before to 5.0 s after this event. This procedure produced a pair of synchronized videos for each riding trial, which was used for subsequent analysis.

### 2.3. Deep Learning-Based Rider Detection and Trajectory Extraction

Manually tracking the rider position in every frame of every riding trial would require considerable effort. Therefore, a custom deep learning-based rider detection model was developed by fine-tuning Ultralytics YOLO11n (Ultralytics v8.4.19). The analysis was performed using Python v3.10.12.

**Figure 4:**
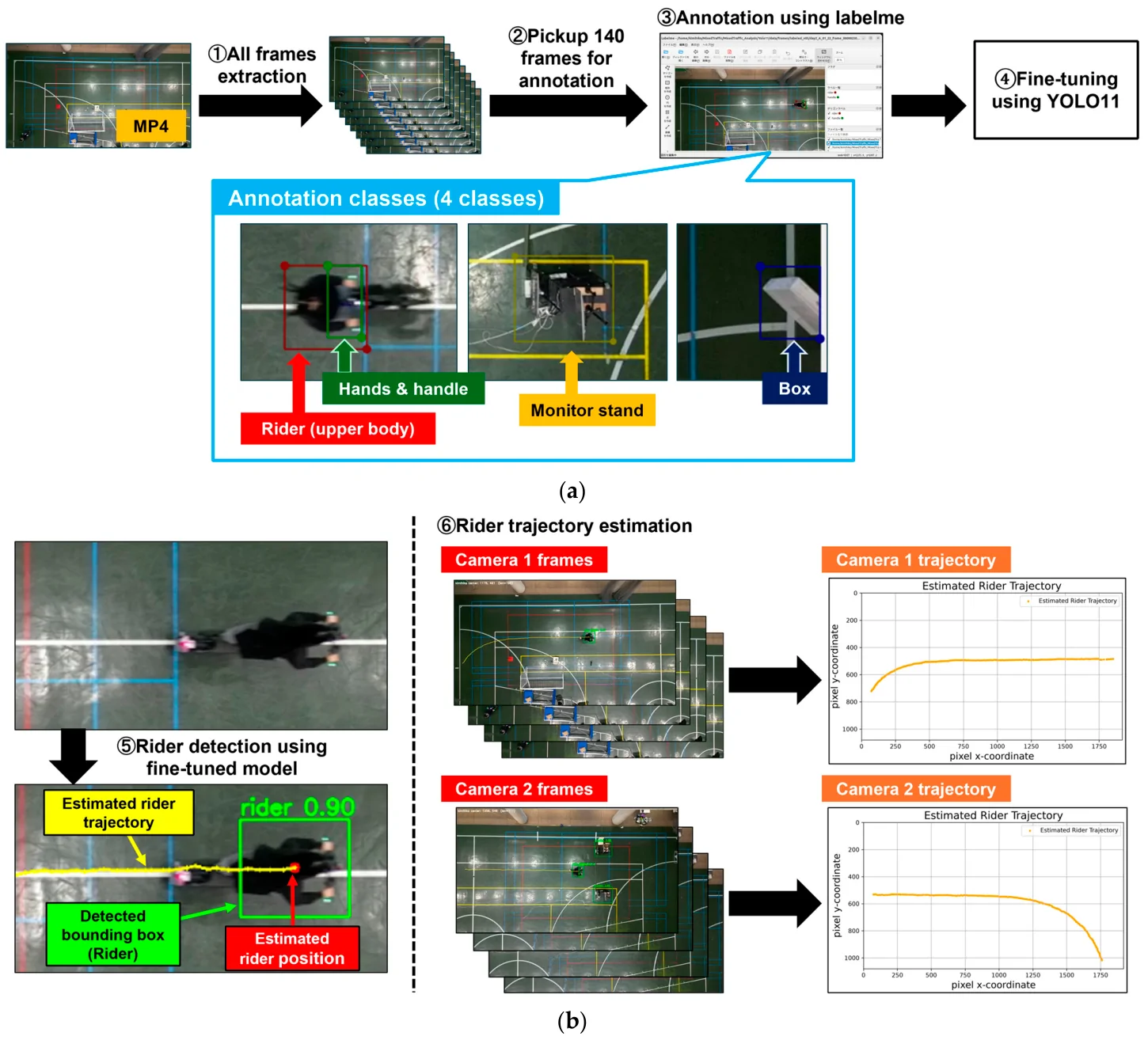
(**a**) Original dataset creation and YOLO11 fine-tuning. (**b**) Rider trajectory estimation.

As shown in Figure 4, 140 frames were extracted from all riding trials to prepare the training dataset. Four classes were manually annotated with bounding boxes using Labelme v5.11.3: “rider,” “hands and handlebar,” “monitor stand,” and “box.” The annotation data were then converted into the YOLO detection format. When only the rider class was used, monitor stands and boxes were occasionally misidentified as riders. Therefore, these objects were defined as separate classes to enable efficient training with a limited number of images.

As shown in Table 2, the input image size was set to 640 × 640 pixels. The batch size was 16, and the maximum number of epochs was 300. The applied data augmentation methods included adjustments to hue, saturation, and brightness. Translation of up to 10%, scaling of up to 50%, horizontal flipping with a probability of 0.5, and Mosaic augmentation were also applied. Vertical flipping, rotation, shearing, perspective transformation, MixUp, CutMix, and Copy-Paste were not applied. The random seed was set to 0, and deterministic execution was enabled. Of the 140 extracted images, 113 images (80.7%) were used for training, and 27 images (19.3%) were used for validation.

As shown in Table 3, the fine-tuned model achieved a precision of 95.6% and a recall of 100% for rider detection.

Even in overhead-view videos, the appearances of the rider and e-scooter differ between the center and periphery of the camera’s field of view. When the entire rider and e-scooter were annotated as a single target, the bounding boxes tended to become horizontally elongated near the image periphery. This resulted in substantial variation in the shape of the detection regions. Therefore, only the rider’s upper body was selected as the detection target. This approach reduced the effect of position-dependent appearance variations and improved detection stability.

The fine-tuned YOLO11n model was applied to every frame of each riding trial. A bounding box representing the rider’s upper-body region was obtained in each frame. The center of the detected bounding box was defined as the rider position. The rider’s trajectory was then obtained as a time series of these positions across consecutive frames. Specifically, let the upper-left coordinates of the bounding box detected in frame t be $\left(x_{min},y_{min}\right)$, and let the lower right coordinates be $\left(x_{max},y_{max}\right)$. Then, the rider position $\left(u_{t},v_{t}\right)$ is given as follows:

(1) $$u_{t}=\frac{x_{min}+x_{max}}{2},v_{t}=\frac{y_{min}+y_{max}}{2},$$

For each riding trial, the rider position was estimated in every frame captured by Camera 1 and Camera 2. The estimated riding trajectory was then obtained separately for each camera.

### 2.4. ORB-Based Image Registration and Trajectory Integration

The riding trajectories from Camera 1 and Camera 2 were integrated to cover the entire riding lane. This required estimation of the geometric relationship between the two camera images. Both trajectories were then represented in a common coordinate system.

ORB features and RANSAC were used for image registration using OpenCV v4.13.0. ORB is relatively robust to rotation and has a low computational cost. It was therefore suitable for matching images captured by hovering drones. RANSAC enabled robust transformation estimation even when the matched points contained outliers.

**Figure 5:**
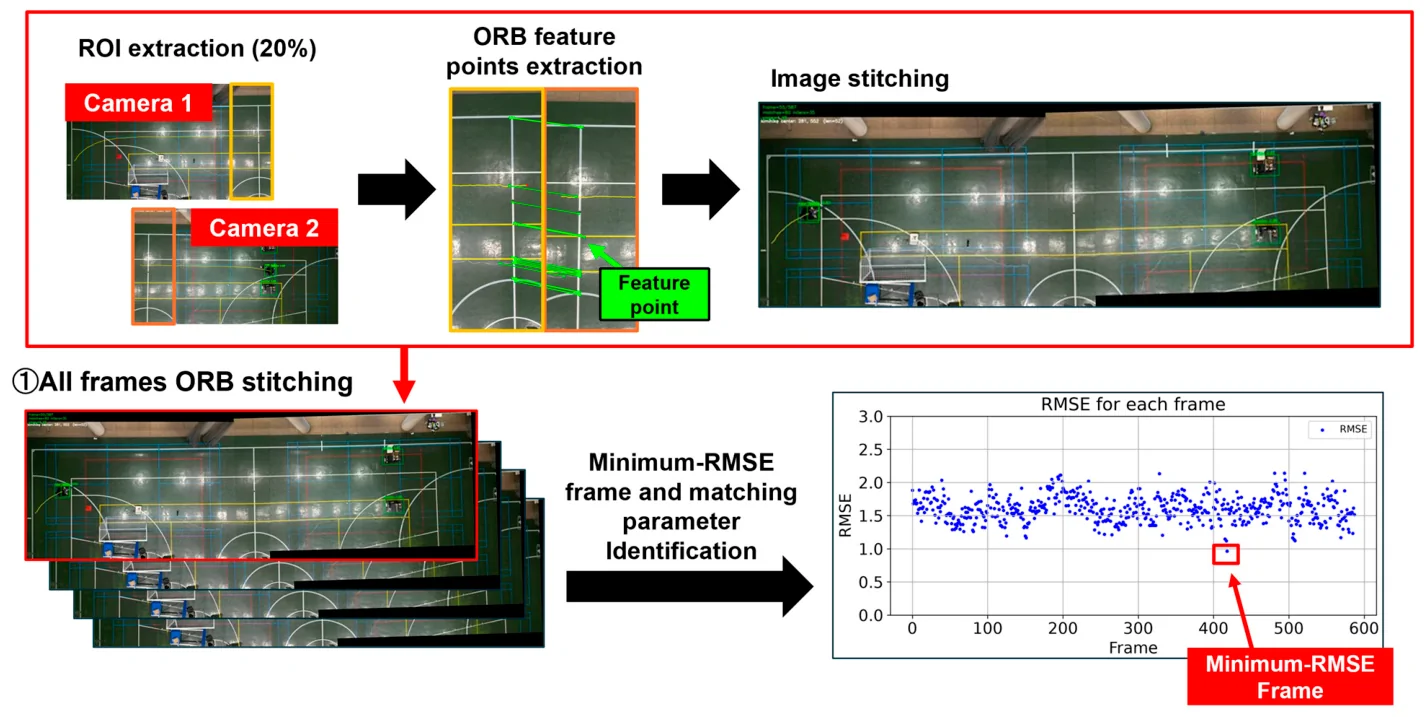
Image stitching using ORB.

Feature extraction and matching were limited to the overlapping regions. As shown in Figure 5, these regions were defined as the rightmost 20% of the Camera 1 image and the leftmost 20% of the Camera 2 image. Feature points and descriptors were extracted only from these regions. This reduced unnecessary feature points from non-overlapping background areas. It also improved matching stability and reduced computational cost.

Each overlapping region was first converted to grayscale. ORB was then used to extract feature points and descriptors. Candidate matches were obtained using a brute-force matcher based on the Hamming distance. Incorrect matches were removed using a distance-ratio test. RANSAC was then applied to the remaining matches. A similarity transformation from Camera 2 to Camera 1 was estimated. The transformation is expressed as follows:

(2) $$\left[\begin{array}{c} u^{\prime }\\ v^{\prime } \end{array}\right]=\left[\begin{array}{cc} a & -b\\ b & a \end{array}\right]\left[\begin{array}{c} u\\ v \end{array}\right]+\left[\begin{array}{c} t_{x}\\ t_{y} \end{array}\right],$$

Here, a and b are coefficients including the rotation and uniform scaling components, and $t_{x}$ and $t_{y}$ are the translation components. Through this transformation, the trajectory position $\left(u_{t}^{\left(R\right)},v_{t}^{\left(R\right)}\right)$ obtained from camera 2 can be converted into camera 1’s image coordinate system.

Furthermore, to quantitatively evaluate the accuracy of image matching, we calculated the root mean square error (RMSE) of the reprojection error for the inlier correspondences obtained by RANSAC. Based on the distance between the predicted point ${\hat{p}}_{i}^{\prime }$, obtained by mapping the corresponding point $p_{i}$ using the transformation matrix M, and the actual corresponding point $p_{i}^{\prime }$, the RMSE is defined as follows:

(3) $$RMSE=\sqrt{\frac{1}{N}\sum_{i=1}^{N}{∥{\hat{p}}_{i}^{\prime }-p_{i}^{\prime }∥}^{2}},$$

Here, N denotes the number of inlier correspondences. A smaller RMSE indicates that the estimated transformation explains the corresponding points more accurately.

For each riding trial, ORB-based image registration and RMSE calculation were performed for every corresponding frame pair. The parameters from the frame pair with the lowest RMSE were used for trajectory integration.

### 2.5. Horizontal Alignment and Metric Conversion

ORB-based image registration and trajectory integration placed the riding trajectories from Camera 1 and Camera 2 in a common image coordinate system. However, the origin and lane orientation still differed among riding trials. The coordinates had also not been converted into real-world distances. Therefore, direct comparisons among riding trials were difficult.

To address this issue, two reference points separated by a known distance were defined on the stitched image. These points were used to translate, rotate, and scale the coordinates. This process produced a common real-world coordinate system.

**Figure 6:**
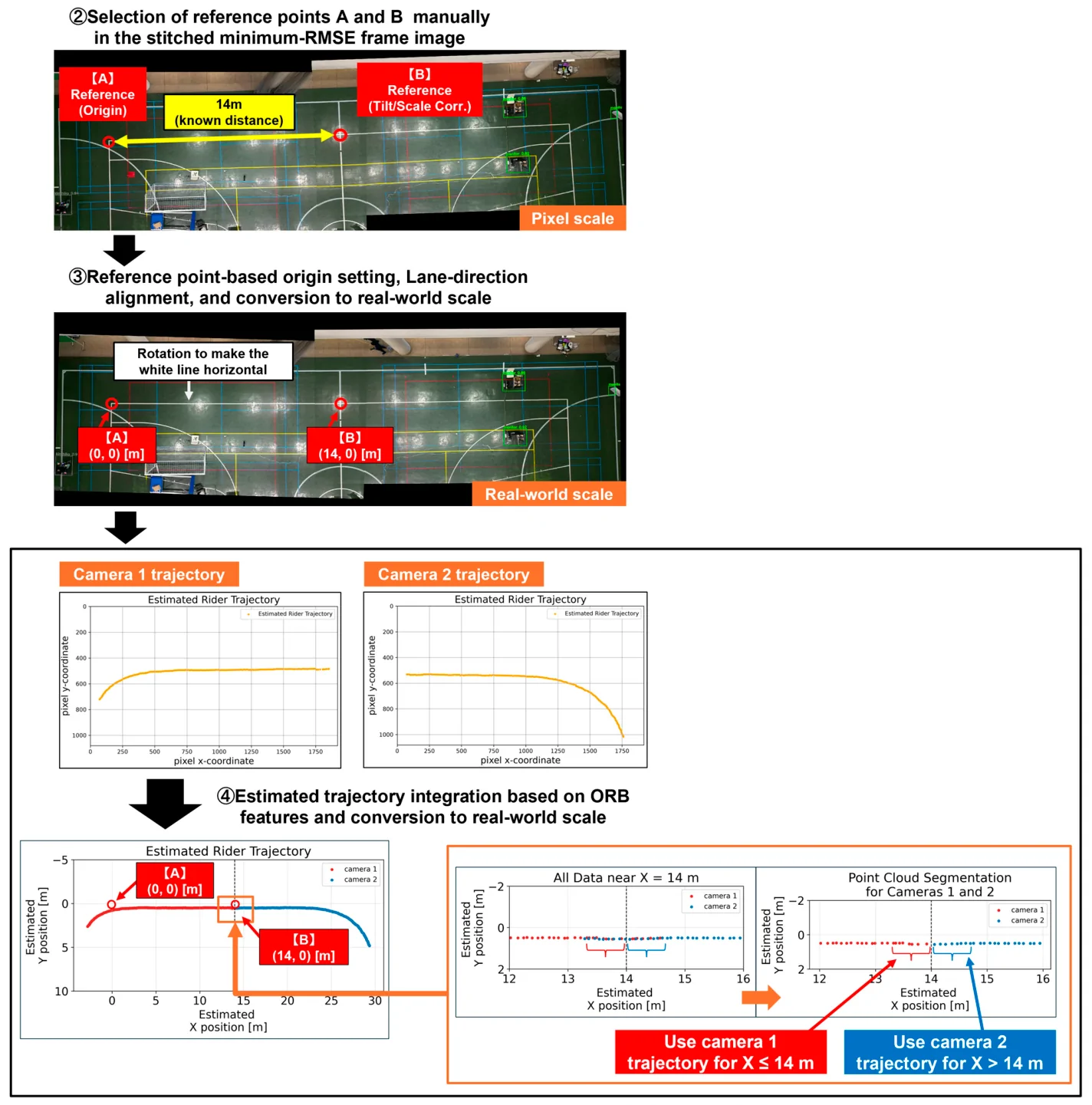
Horizontal alignment and metric conversion using reference points with a known distance, and trajectory integration between Cameras 1 and 2.

As shown in Figure 6, reference points A and B were defined on the stitched image. Based on the standard dimensions of the basketball court in the gymnasium, the known distance between A and B was 14 m. Point A was defined as the origin. Point B defined the positive direction of the x-axis along the riding lane. The riding trajectories from Camera 1 and Camera 2 were integrated using the image-registration parameters with the lowest RMSE obtained in Section 2.4.

All trajectory coordinates were translated so that Point A coincided with the origin. The translated coordinates were then rotated to align line segment AB with the x-axis. Let the coordinates of point A be $\left(u_{A},v_{A}\right)$, the coordinates of point B be $\left(u_{B},v_{B}\right)$, and an integrated trajectory point be $\left(u,v\right)$. Then, the translated coordinates $\left(u^{\prime },v^{\prime }\right)$ are given as follows:

(4) $$u^{\prime }=u-u_{A},v^{\prime }=v-v_{A},$$

Furthermore, using the angle formed by line segment AB,

(5) $$\theta =atan\;2\left(v_{B}-v_{A},u_{B}-u_{A}\right),$$

The coordinates after horizontal alignment $\left(x,y\right)$ were obtained by rotating the coordinates by −θ. This transformation is expressed as follows:

(6) $$\left[\begin{array}{c} x\\ y \end{array}\right]=\left[\begin{array}{cc} \cos\theta & \sin\theta \\ -\sin\theta & \cos\theta \end{array}\right]\left[\begin{array}{c} u^{\prime }\\ v^{\prime } \end{array}\right],$$

Furthermore, the normalized image coordinates were converted into real-world coordinates in meters. Let the coordinates of points A and B after horizontal alignment be $\left(x_{A},y_{A})=(0,0\right)$ and $\left(x_{B},y_{B}\right)$, respectively. Then, the distance between A and B in the image coordinate system, $d_{AB}$, is given as follows:

(7) $$d_{AB}=\sqrt{\left(x_{B}-x_{A}{)}^{2}+(y_{B}-y_{A}{)}^{2}\right.},$$

Here, when the real distance between A and B is denoted by $L_{AB}=14\;\left[m\right]$, the conversion factor from image coordinates to real-world coordinates, s[m/pixel], is given as follows:

(8) $$s=\frac{L_{AB}}{d_{AB}},$$

Accordingly, an arbitrary trajectory position $\left(x,y\right)$ after horizontal alignment is expressed in the real-world coordinate system $\left(X,Y\right)$ with A as the origin, as follows:

(9) X=sx,Y=sy,

The riding trajectories from both cameras overlapped near Point B, located 14 m from the origin along the x-axis. Point B was defined as the inter-camera junction and used as the switching boundary. The Camera 1 trajectory was used for positions up to and including Point B. The Camera 2 trajectory was used for positions beyond Point B.

### 2.6. Experimental Setup

As shown in Table 4, seven riders participated in the experiment. Each rider carried a shoulder bag while riding along the lane, as shown in Figure 1. A staff member was stationed at the starting point and signaled permission to start. Each rider then began the riding trial at a self-selected time.

After two practice trials, a total of 24 riding trials were conducted. Other measurements were performed simultaneously during the experiment. The latter 12 trials were recorded using the proposed multi-camera video measurement system.

Each drone recorded the trials using its automatic hovering function. Due to the recording specifications, each video was saved as a separate file approximately every 3 min 17 s. Riding trials that extended across two video files were excluded from the analysis. Videos in which a drone rotated substantially during a riding trial were also excluded.

### 2.7. Evaluation Metrics

**Figure 7:**
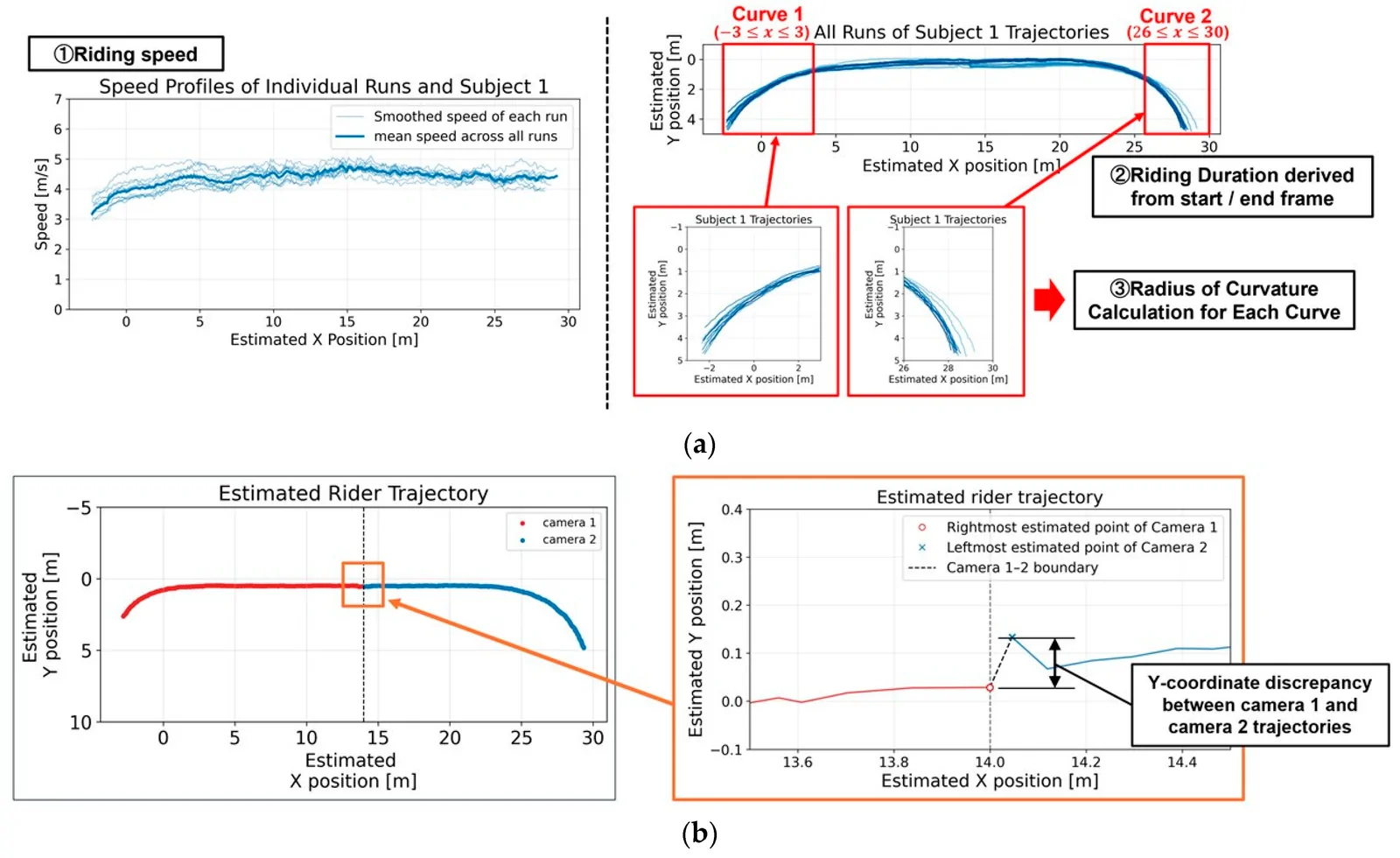
(**a**) Rider behavior comparison metrics. (**b**) Inter-camera junction discrepancy in the y-direction.

As shown in Figure 7a, three metrics were calculated to compare riding behavior among subjects: riding speed, riding duration, and the radius of curvature for each of the two curves in the riding lane.

The riding data were analyzed at intervals of 0.1 m. A moving average with a window size of 20 was applied for smoothing, and the riding speed was calculated. The riding duration was calculated from the frame count and video frame rate of each riding trial.

The region from −3 to 3 m along the x-axis was defined as Curve 1. The region from 26 to 30 m was defined as Curve 2. The radius of curvature was calculated for each curve by applying a circle-fitting procedure implemented in SciPy to the riding trajectory within each region.

As shown in Figure 7b, the inter-camera junction discrepancy was calculated to quantify the consistency of multi-camera trajectory integration. The position at 14 m along the x-axis was defined as the inter-camera junction. The absolute difference between the y-coordinates immediately before and after the junction was used as the inter-camera junction discrepancy.

## 3. Results

**Figure 8:**
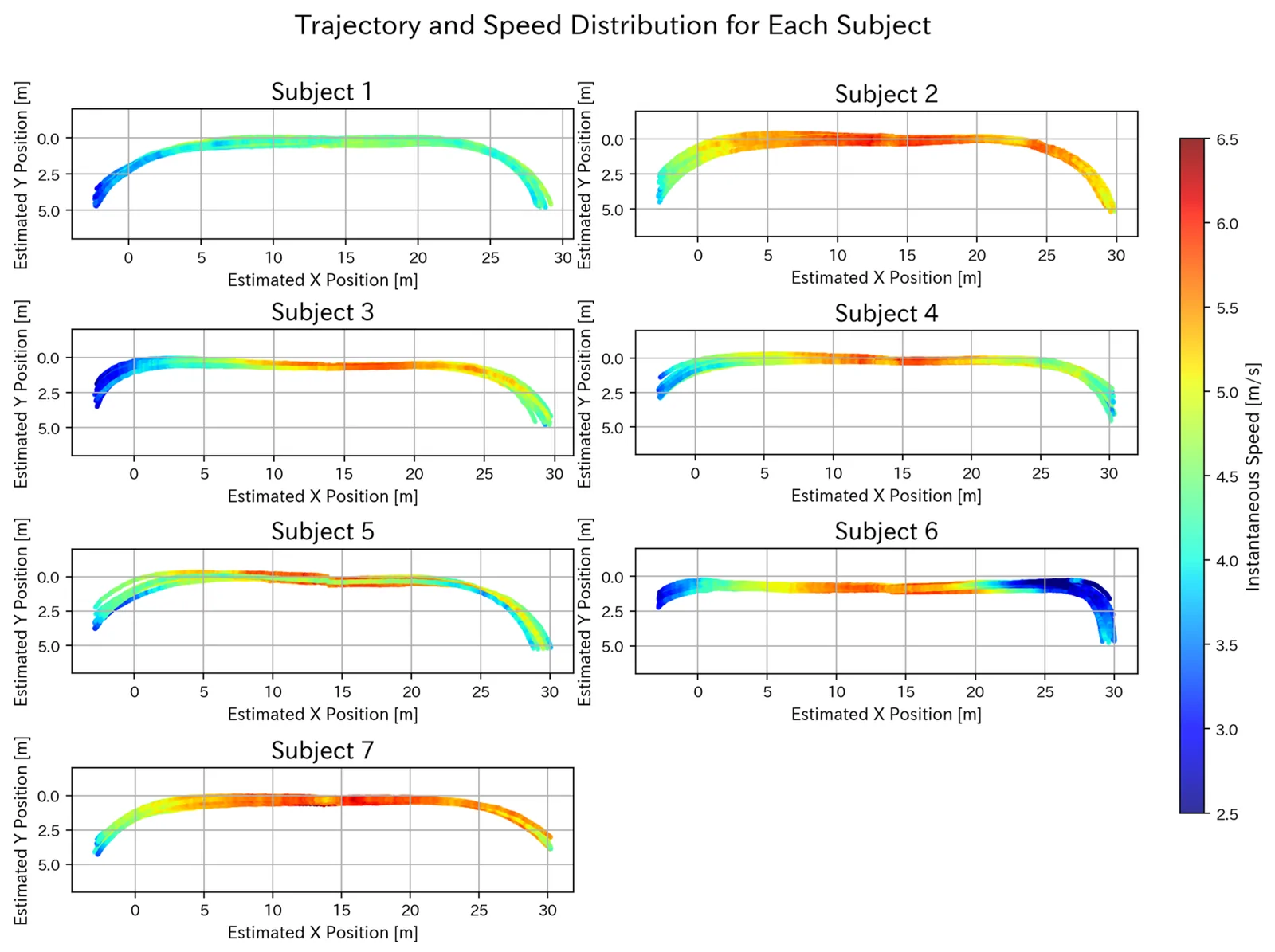
Trajectories and speed distributions based on the estimated positions of each subject.

Figure 8 shows the riding position and speed of each subject. The plotted values were smoothed using a moving average filter with a window size of 20 frames. No reference trajectory was prescribed in this experiment. The subjects were allowed to ride freely within the riding lane. Subject 1 showed a lower speed in the straight section than Subjects 2–7. Subject 6 decelerated more before entering the curve than the other subjects. These results indicated differences among subjects in both riding trajectory and speed control.

**Figure 9:**
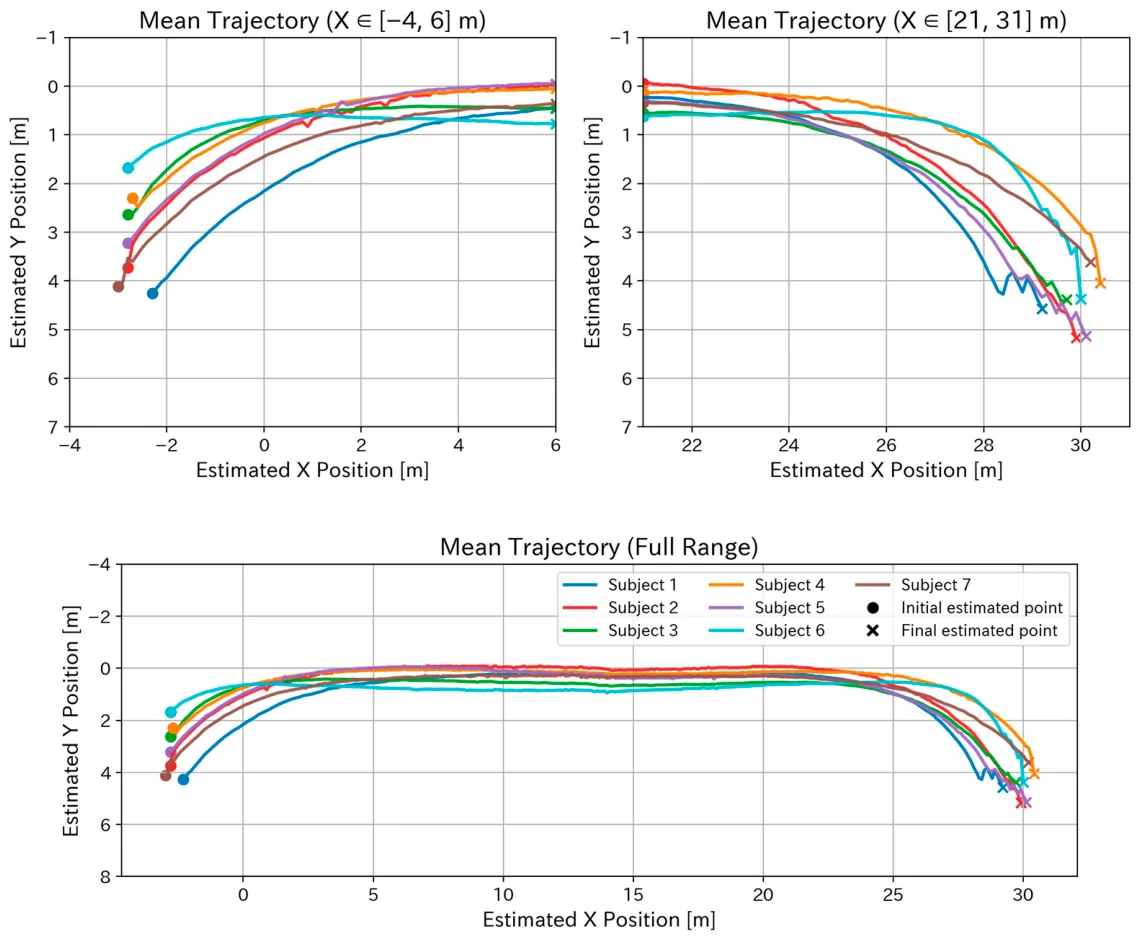
Average riding trajectories based on the estimated positions of each subject (averaged at 0.1 m intervals in the x-direction).

**Figure 10:**
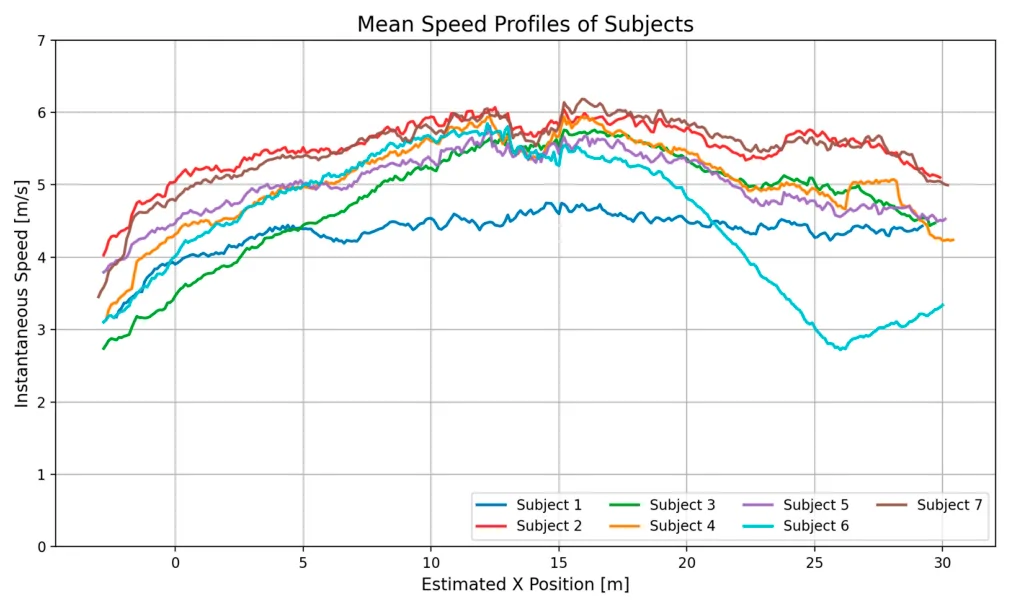
Average riding speeds based on the estimated positions of each subject (averaged at 0.1 m intervals in the x-direction).

For each subject, the riding trajectory and speed were averaged at intervals of 0.1 m along the x-axis using all riding trials. The average riding trajectories are shown in Figure 9, and the average riding speeds are shown in Figure 10. The plotted values were smoothed using the same moving average filter with a window size of 20 frames. Figure 9 shows differences in the representative riding trajectories among subjects. Subject 1 followed a pronounced inward-curving trajectory. In contrast, Subjects 4 and 6 followed trajectories that maintained longer straight sections.

The average speed profiles shown in Figure 10 were generally consistent with the trends observed in Figure 8. Subject 1 had the lowest riding speed in the straight section. Subject 6 showed a marked decrease in speed during the latter half of the straight section. Before turning, Subject 6 decelerated to approximately half of the maximum speed.

**Figure 11:**
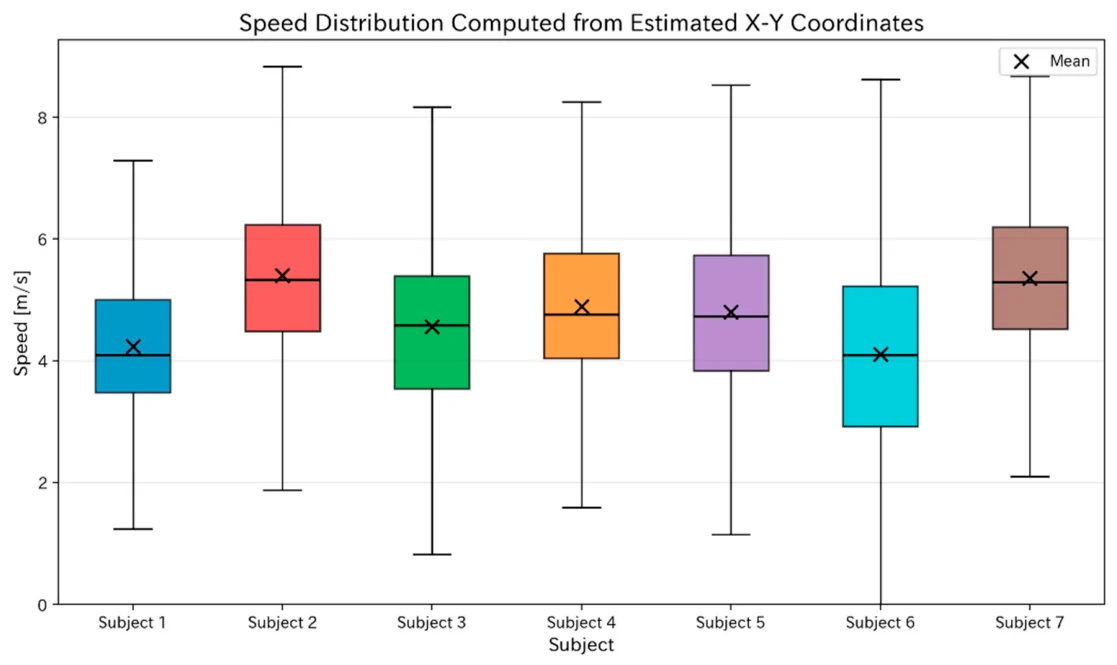
Speed distributions derived from the estimated positions of each subject.

**Figure 12:**
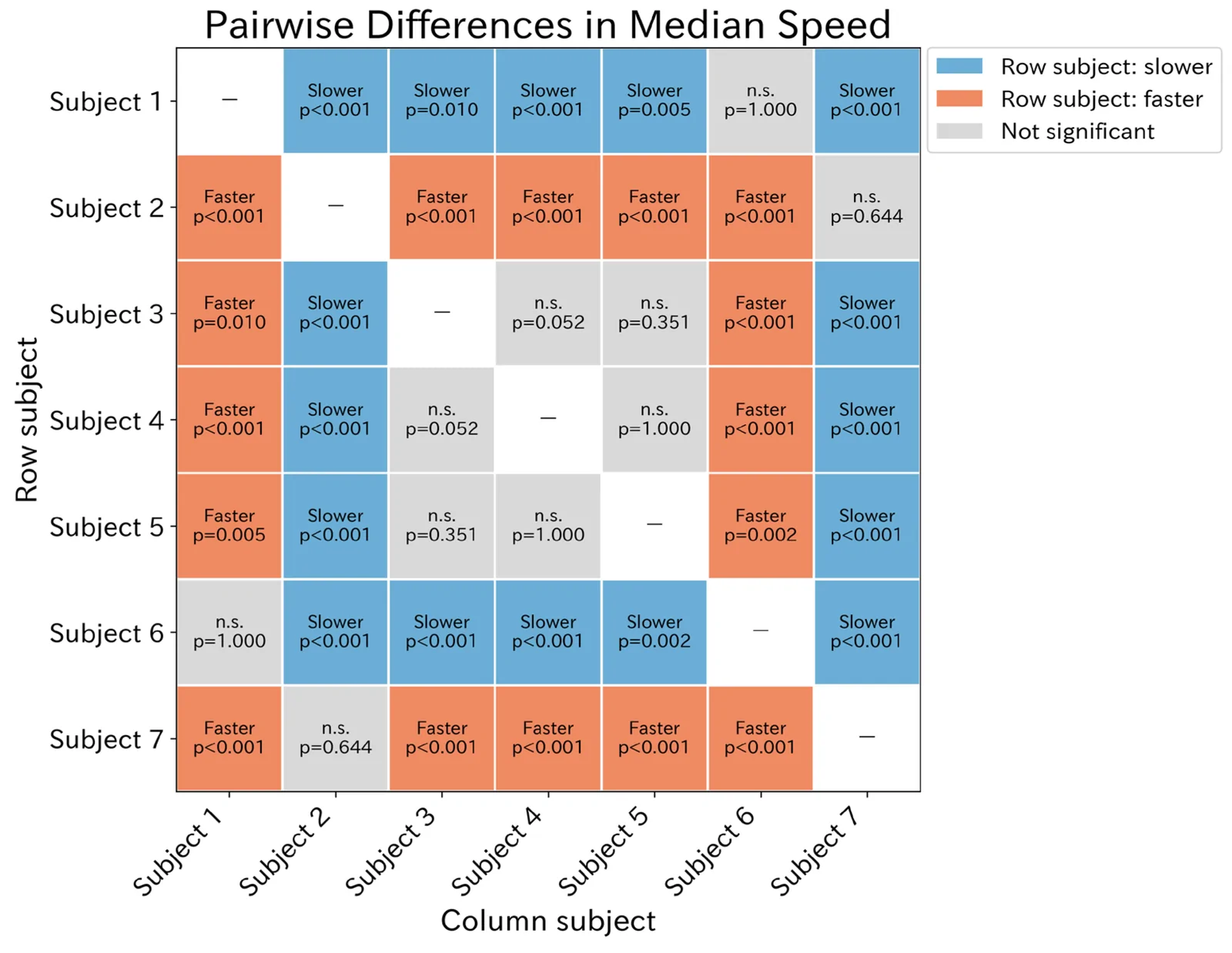
Pairwise two-sided Mann–Whitney U tests for riding speed among subjects.

The speed distributions calculated from the estimated positions are shown in Figure 11 and Table 5. The quantitative results were consistent with the trends described above. Subject 6 had the lowest average speed at 4.1 m/s. Subject 1 had the lowest third-quartile value. The results of the pairwise two-sided Mann–Whitney U tests for riding speed among subjects are shown in Figure 12.

**Figure 13:**
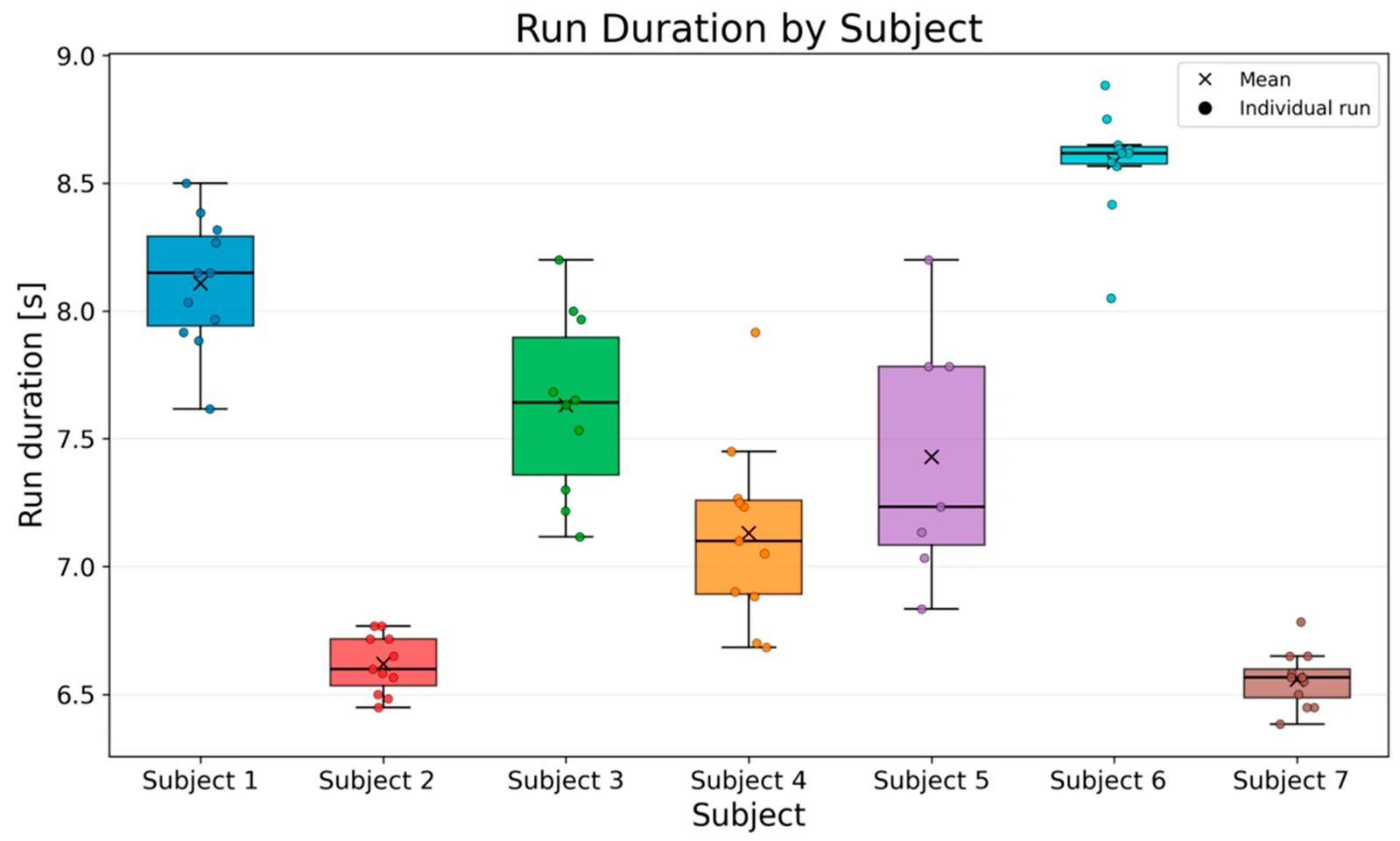
Riding duration per trial.

**Figure 14:**
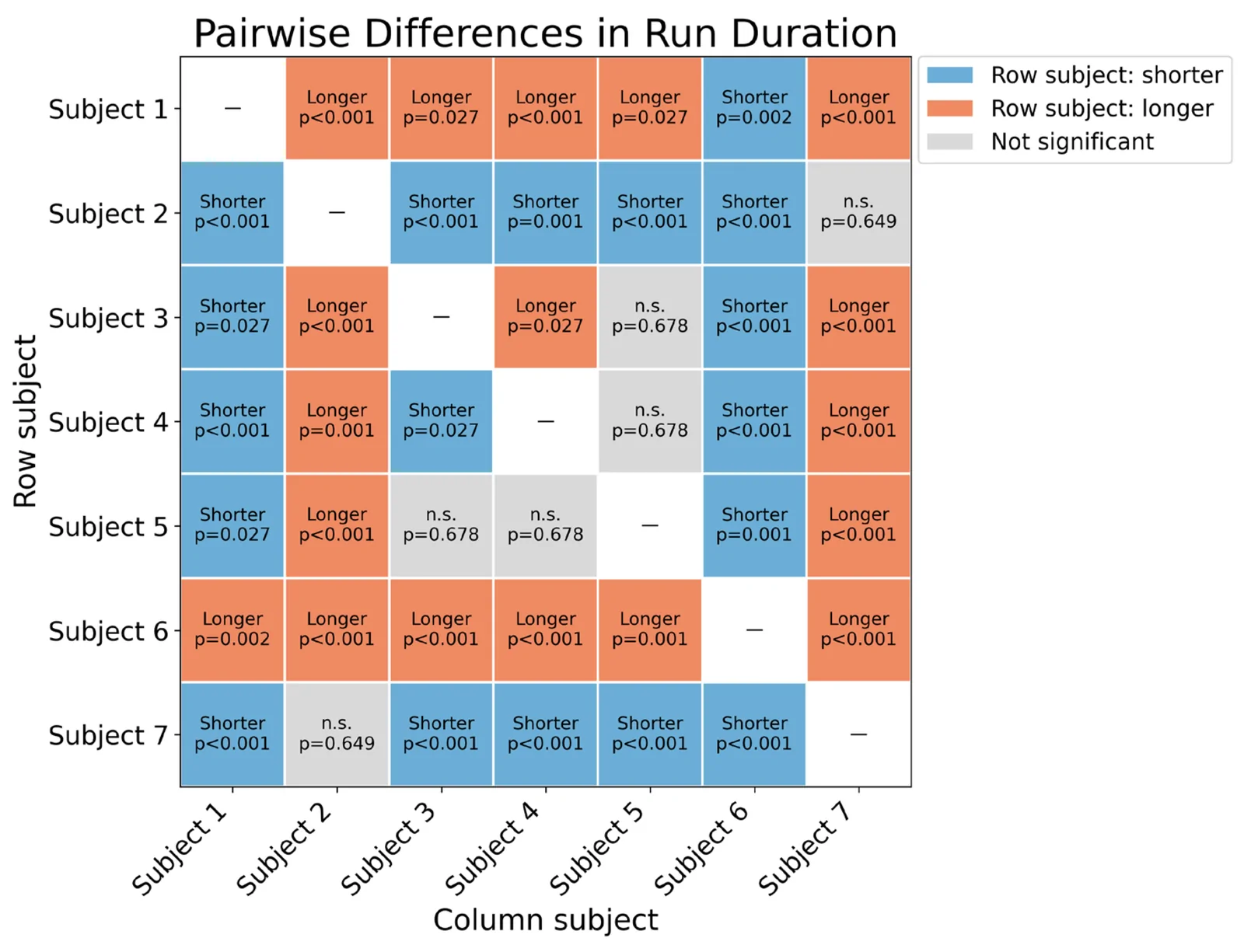
Pairwise two-sided Mann–Whitney U tests for riding duration among subjects.

The riding duration is shown in Figure 13 and Table 6. Subjects 2, 6, and 7 showed less variation in riding duration than the other four subjects. Subjects 2 and 7 had shorter riding durations of approximately 6.5 s. Subject 6 had the longest riding duration at 8.5 s. The results of the pairwise two-sided Mann–Whitney U tests for riding duration among subjects are shown in Figure 14.

**Figure 15:**
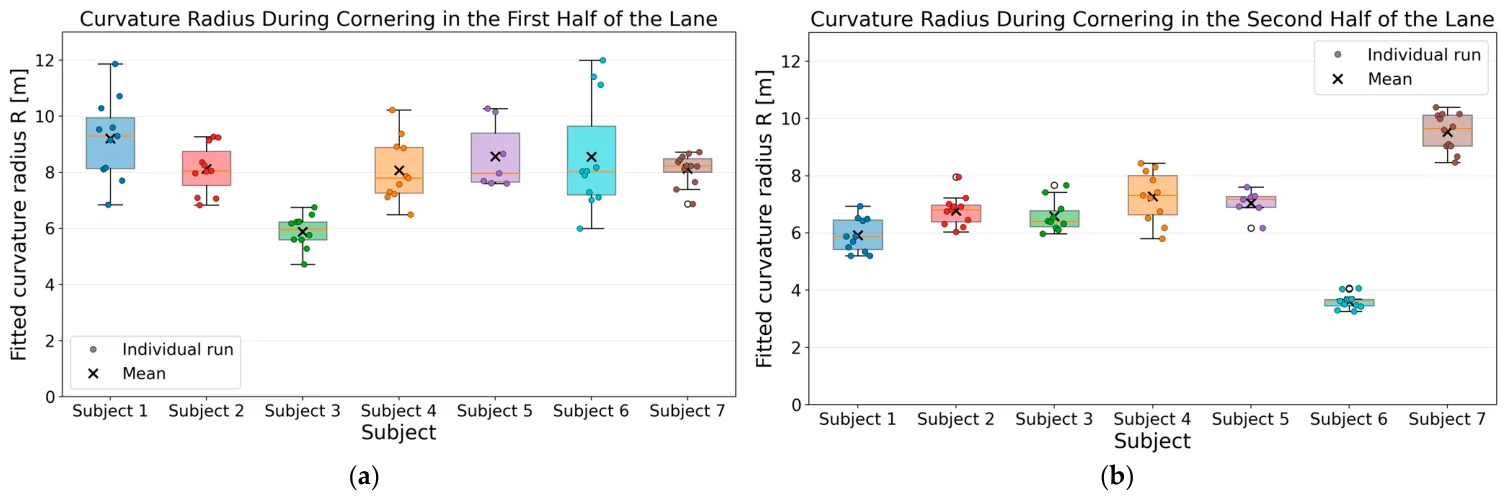
(**a**) Radius of curvature for Curve 1. (**b**) Radius of curvature for Curve 2.

**Figure 16:**
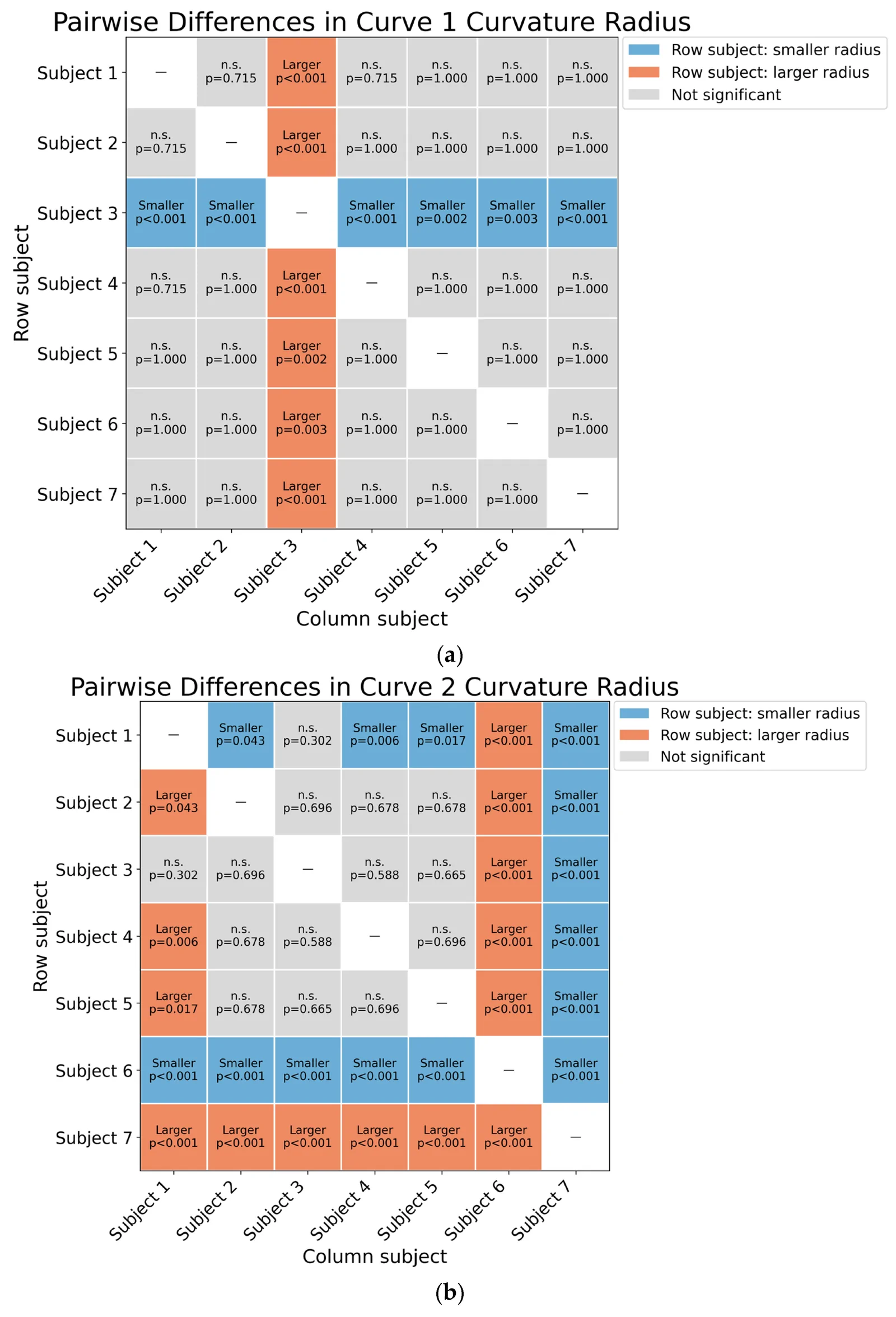
(**a**) Pairwise two-sided Mann–Whitney U tests for the radius of curvature for Curve 1 among subjects. (**b**) Pairwise two-sided Mann–Whitney U tests for the radius of curvature for Curve 2 among subjects.

Figure 15 and Table 7 and Table 8 show the radius of curvature for Curves 1 and 2. For the curve immediately after the start, Subject 3 had the smallest radius of curvature. No substantial differences were observed among the other subjects. For the curve in the latter section of the riding lane, Subject 6 had the smallest radius of curvature at 3.6 m. Subject 7 had the largest radius at 9.5 m. The riding trajectories in Figure 8 and Figure 9 show the same trends. The results of the pairwise two-sided Mann–Whitney U tests for the radius of curvature among subjects are shown in Figure 16.

Pairwise two-sided Mann–Whitney U tests were conducted for all pairs of subjects at a significance level of α = 0.05. The tests were applied to the distributions of riding speed shown in Figure 11, riding duration shown in Figure 13, and the radius of curvature for each curve shown in Figure 15. 

**Figure 17:**
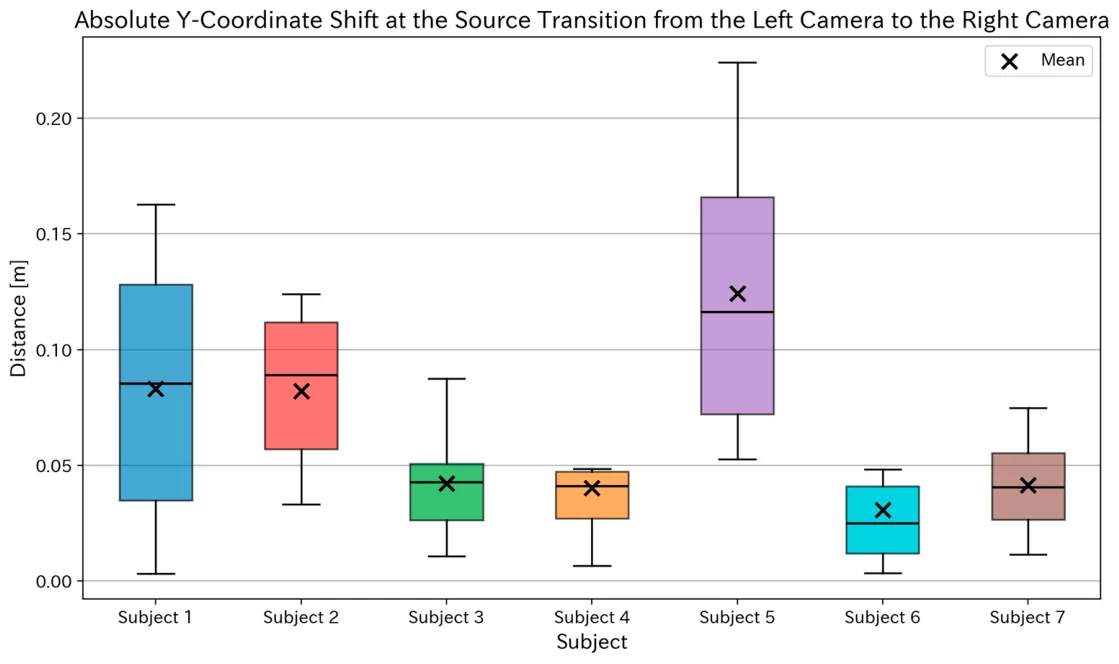
Distribution of the inter-camera junction discrepancy in the y-direction.

Figure 17 and Table 9 show the inter-camera junction discrepancy. This metric was calculated as the absolute difference between the estimated y-coordinates in the final frame of Camera 1 and the initial frame of Camera 2. Subject 5 had a mean discrepancy of 12.4 cm. The mean discrepancy was within 10 cm for all other subjects. It was within 5 cm for Subjects 3, 4, 6, and 7.

## 4. Discussion

No predefined reference trajectory was specified in this experiment. Subjects were allowed to ride freely within the riding lane. As shown in Figure 8, subject-specific riding behaviors were observed, including low-speed riding, high-speed riding, and deceleration before turning. The average riding trajectories also differed among the subjects. Subject 1 followed a trajectory extending farther toward the inside of the lane. In contrast, Subjects 4 and 6 maintained longer straight sections. Similar trajectory characteristics were qualitatively observed across multiple riding trials for Subject 1. These results suggest that the proposed method can capture subject-specific riding trajectory characteristics.

For the quantitative evaluation, three measures were calculated: (1) riding speed, (2) riding duration per trial, and (3) the radii of curvature of the curves in the first and second halves of the riding lane. Differences in these measures among the subjects were then examined.

Regarding riding speed, Subject 1 had a lower maximum speed in the straight section and a lower mean speed, as shown in Table 5. As shown in Figure 10, the speed of Subject 1 remained between 4 and 5 m/s beyond approximately 5 m from the starting position. This result indicates low-speed riding with limited acceleration and deceleration. In contrast, Subjects 2 and 7 maintained relatively high speeds throughout the riding trials. Their speed distributions were generally shifted toward higher values. These subjects therefore appeared to continue riding without substantial deceleration after acceleration.

The speed of Subject 6 decreased markedly in the latter half of the straight section and reached its minimum immediately before entering the curve. This behavior may reflect anticipatory deceleration in preparation for turning rather than merely reactive deceleration. The test results in Figure 12 showed that the speed distribution of Subject 1 was significantly lower than that of Subject 6. However, the deceleration of Subject 6 in the latter half of the riding lane likely reduced the mean speed. Thus, the two subjects exhibited different characteristics. Subject 1 rode at a low speed throughout the riding lane, whereas Subject 6 decelerated markedly before the curve. The speed distributions of Subjects 2 and 7 were also significantly higher than those of the other subjects.

Regarding riding duration, Subjects 2, 6, and 7 had narrower interquartile ranges than the other four subjects. This result indicates less variability among their riding trials. The test results in Figure 14 showed that the riding durations of Subjects 2 and 7 were significantly shorter than those of the other subjects. In contrast, Subject 6 had a significantly longer riding duration and the narrowest interquartile range. A narrow interquartile range indicates consistent riding duration across trials. Therefore, riding duration and its variability may distinguish subjects who ride consistently at high speeds from those who ride consistently at low speeds.

Regarding the radius of curvature, Subject 3 showed a smaller radius in Curve 1, located in the first half of the riding lane. This result indicates a tendency to make a tighter turn. However, no significant differences were found among the subjects for Curve 1. In Curve 2, located in the second half of the riding lane, Subject 6 had a significantly smaller radius of curvature, indicating a tighter turn. Subject 7 had a significantly larger radius, indicating a wider turn. These results suggest that the radius of curvature can capture subject-specific differences in turning strategies.

The course used in this experiment had a simple curve–straight–curve layout. Future studies should evaluate the proposed method using more complex layouts that include slalom sections and obstacles.

Overall, this study demonstrated that riding speed, the variability in riding duration, and the radius of curvature can be used to distinguish subject-specific riding characteristics. These measures enabled quantitative assessment of differences in riding speed, consistency across riding trials, and turning strategies.

As shown in Figure 10, a temporary decrease in speed was observed near Point B (x = 14 m), which was defined as the inter-camera junction between Camera 1 and Camera 2. One possible cause of the increased inter-camera junction discrepancy was unstable detection of the subject’s upper body. The center of the detected upper-body bounding box was defined as the estimated rider position. However, when the detection target moved from the center toward the periphery of the camera view, the bounding box became wider in the horizontal direction. This change may have affected the estimated position. A contrast in clothing color between the upper and lower body may help stabilize upper-body detection.

**Figure 18:**
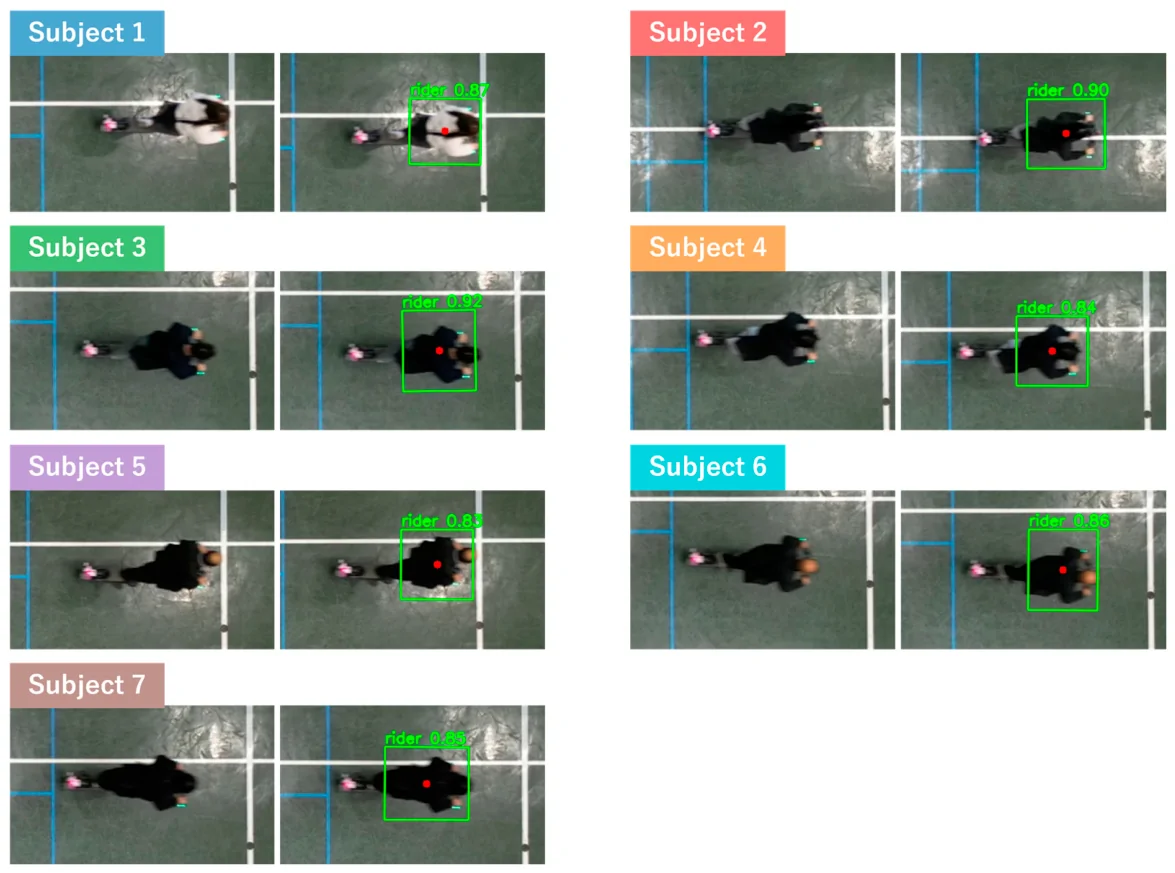
Clothing of each subject.

As shown in Figure 18, Subject 1 wore white clothing on the upper body and black clothing on the lower body. This color contrast may have stabilized upper-body detection. Consequently, no clear decrease in speed was observed for Subject 1 at the inter-camera junction.

As shown in Figure 17, the inter-camera junction discrepancy in the y-coordinate also varied among the subjects. Subject 5 had a mean discrepancy of 12.4 cm, which was larger than those of the other subjects. In contrast, the mean discrepancies for Subjects 3, 4, 6, and 7 were within 5 cm. These results indicate that the proposed trajectory integration was generally consistent under the present experimental conditions. However, the discrepancy may increase under specific conditions.

One possible reason for the larger discrepancy observed for Subject 5 was that the subject rode slightly diagonally through the straight section. Most riding trajectories near the inter-camera junction were nearly straight and aligned with the x-axis. However, diagonal riding trajectories may occur in experiments involving obstacles or factors that induce deceleration. Therefore, the trajectory-matching method in the overlapping region should be improved in future work.

In the proposed method, the center of the upper-body bounding box was used as the rider position in each frame. This approach assumes that the upper-body position provides a stable proxy for the ground-plane position of the e-scooter. However, changes in posture during turning, upper-body lean, and the position within the camera view may shift the center of the bounding box. Such changes may appear in the estimated riding trajectory and speed as apparent displacements unrelated to the actual movement of the e-scooter. Therefore, the position obtained by the proposed method represents an observed representative position of the rider. It does not directly represent the absolute ground-plane position of the e-scooter. This limitation should be considered when analyzing small posture changes or subtle variations in the riding trajectory.

In this experiment, only one subject rode during each riding trial, and multiple riders were not simultaneously present in the videos. Consequently, occlusion and overlap between riders did not occur, and neither multi-object tracking nor cross-camera identity association was required. The trajectories obtained from Cameras 1 and 2 were extracted from synchronized videos of the same riding trial. The current platform is therefore intended for repeated riding evaluation involving one rider at a time. Application to environments involving multiple simultaneous riders will require a multi-object tracking method capable of handling occlusion and rider overlap while maintaining rider identities across cameras.

Lens-distortion correction is required for higher-precision trajectory tracking and more detailed analysis of riding behavior. A calibration framework is also needed to determine the camera positions and orientations and the relationship between image and real-world coordinates. This calibration should use reference points measured with a high-precision instrument. Furthermore, the estimated positions should be compared with reference positions obtained using an independent measurement system. This comparison is necessary to validate the estimated absolute positions.

Multiple drones were used to capture the entire long and narrow indoor riding lane. The walls and ceiling were close to the drones. Therefore, an automatic tracking function could not be used safely in the present experimental environment. Each drone was instead operated in a fixed hovering position. However, a single drone could capture only part of the riding lane while maintaining sufficient image resolution.

With only one drone, Curve 1 immediately after the start and Curve 2 after the straight section could not be compared within the same riding trial. Although the two curves had similar geometries, the approach conditions differed. These differences may produce different deceleration and turning characteristics. The multi-camera configuration enabled continuous evaluation of these section-specific riding behaviors as a single riding trajectory. Thus, combining multiple drones is a practical option for analyzing riding behavior over a long riding lane. However, increasing the number of drones also increases the complexity of video synchronization, camera calibration, and trajectory integration. More stable methods for calibration, synchronization, and integration should therefore be developed.

Representative drone-based trajectory datasets include highD, which contains vehicle trajectories on highways, and inD and pNEUMA, which contain comprehensive trajectories of road users and urban traffic. These datasets are based on outdoor traffic observations. They are valuable for analyzing naturalistic traffic behavior. However, standardized environmental conditions and repeated riding tasks are important for safety evaluations of individual riding characteristics and behavioral responses to road signs.

Optical motion-capture systems can provide high-precision indoor position measurements. However, such systems are expensive and require cameras to be arranged around the measurement target. Extending the measurement volume to cover a long riding lane therefore places substantial constraints on the experimental setup. The proposed method does not provide the same absolute-position measurement performance as optical motion capture. However, its measurement range can be extended by adding overhead camera coverage without surrounding the entire riding lane with fixed cameras. This is a practical advantage for repeated riding experiments on long indoor riding lanes. In addition, the primary acquisition hardware required for position estimation consists of drones. The system can therefore be deployed in gymnasiums, which are widely available and provide broadly comparable indoor experimental spaces.

The proposed platform provides a fundamental method for repeatedly measuring e-scooter riding behavior in a controlled indoor environment. It can be used to evaluate subject-specific riding characteristics and changes caused by experimental conditions. However, this study was a preliminary evaluation involving only seven subjects and a simple indoor course. The number of subjects, diversity of riding environments, and generalizability to real-world traffic environments were limited. Future studies should include more subjects and more complex riding tasks. Such tasks may involve road signs, traffic signals, obstacles, slalom sections, and other road users. It is also necessary to examine whether the riding characteristics observed indoors are reproduced in real-world environments. This work will support an evaluation framework that combines controlled experiments with observations in naturalistic traffic environments.

## 5. Conclusions

The system design aimed to reduce the effort required for on-site deployment and operation by combining simple hardware with image-analysis-based measurement. Based on this concept, this study developed a multi-camera system for analyzing e-scooter riding trajectories on a long and narrow indoor riding lane. Using videos captured by two cameras, the proposed method extracted rider positions and integrated the resulting trajectories into a common coordinate system. The riding speed distributions and trajectory characteristics were then quantitatively analyzed. The resulting video-based platform enabled non-contact and non-invasive measurement and quantitative analysis of observable riding behavior without requiring subjects to wear sensors.

Rider detection was performed using a deep learning model fine-tuned with images acquired in the experimental environment. The model could detect the rider’s upper body using a relatively small training dataset. However, the dataset used in this study was limited in size. Detection performance may vary with the subject’s clothing, body size, posture, and position within the camera view. Future work should expand the dataset to include diverse subject characteristics, clothing, postures, lighting conditions, and camera viewing angles. Detection performance should also be evaluated under each condition. These improvements will increase detection robustness and reduce measurement bias caused by subject appearance and imaging conditions. They will also improve the comparability of riding behavior among subjects.

The experimental results showed differences among subjects in both riding trajectories and changes in riding speed. The observed characteristics included stable low-speed riding, maintenance of relatively high speeds, and deceleration before entering a curve followed by reacceleration in the subsequent straight section. The average riding trajectories also revealed differences in turning strategies. These included avoiding sharp turns and taking a deeper line through a curve to maintain a longer straight section. These results suggest that the proposed method can characterize subject-specific differences in riding behavior, even under free-riding conditions.

The consistency of trajectory integration between Camera 1 and Camera 2 was evaluated using the inter-camera junction discrepancy in the y-direction. Subject 5 showed a mean discrepancy of 12.4 cm. However, the mean discrepancy was within 10 cm for most subjects and within 5 cm for Subjects 3, 4, 6, and 7. These results indicate that the proposed method achieved generally consistent trajectory integration under the present experimental conditions.

The drone positions were manually adjusted by visually inspecting the images displayed on the controllers. Therefore, slight variations in camera position and viewing angle occurred among flights. Nevertheless, trajectory integration and speed analysis could be performed under these conditions. Temporary decreases in speed and increased inter-camera junction discrepancies were observed near the inter-camera junction. Variations in bounding-box shape caused by the camera viewing angle and subject posture may have contributed to these discrepancies.

The estimated trajectories were not compared with independently measured reference positions. Therefore, the absolute positional validity of the proposed system could not be determined. The inter-camera junction discrepancy reported in this study should not be interpreted as a measure of absolute trajectory validity. Instead, it represents the internal consistency of trajectory integration between the two cameras. The lack of independent reference-based validation is a limitation of this study and should be addressed in future work.

Overall, the proposed system may provide a useful method for quantitatively characterizing e-scooter riding trajectories and speed characteristics on an indoor riding lane. It also enables comparisons of riding behavior among subjects. Future work should expand the training dataset, improve trajectory matching at the inter-camera junction, and introduce a position-estimation method that is robust to posture changes. The estimated absolute positions should also be validated using an independent measurement system. Furthermore, the system should be evaluated under diverse experimental conditions, including tasks involving obstacles and induced deceleration. These improvements will enable more precise and comprehensive analysis of micromobility riding behavior.

## Figures and Tables

**Table 1:** Camera Specifications of the DJI Mini 3 Pro.

Parameter	Specification
Image sensor	1/1.3-inch CMOS
Effective pixels	48 MP
Lens	24 mm (35 mm format)
Field of view (FOV)	82.1°
Aperture	f/1.7
Video resolution/frame rate	4K/60 fps
Camera angle	Nadir

**Table 2:** YOLO11 fine-tuning settings.

Items	Details
Model	YOLO11n
Input image size	640 × 640 pixels
Batch size	16
Epochs	300
Annotation class	4 (rider, hands & handle, monitor stand, box)
Dataset (images)	Training: 113 (80.7%)/Validation: 27 (19.3%)

**Table 3:** YOLO11 fine-tuning result.

Class	Precision	Recall
Rider	0.956	1.00
Hands & handle	0.989	0.88
Monitor stand	0.982	1.00
Box	0.964	1.00

**Table 4:** Subject configuration.

Subject	Age	Sex	Driver’s License	E-Scooter Experience
1	19	Female	-	-
2	19	Female	-	-
3	19	Male	Car	-
4	19	Male	Car	-
5	50	Male	Car	-
6	63	Male	Car/Bus	-
7	21	Male	Car/Motorcycle	~ once every 6 months

**Table 5:** Summary of the descriptive statistics of speed for each subject.

Subject	Subject 1	Subject 2	Subject 3	Subject 4	Subject 5	Subject 6	Subject 7
Mean [m/s]	4.234	5.401	4.558	4.89	4.805	4.105	5.358
Q1 [m/s]	3.48	4.489	3.541	4.041	3.844	2.923	4.528
Q2 [m/s]	4.091	5.325	4.584	4.757	4.725	4.086	5.289
Q3 [m/s]	5.007	6.232	5.393	5.765	5.731	5.227	6.195

**Table 6:** Summary of riding duration per trial.

Subject	Subject 1	Subject 2	Subject 3	Subject 4	Subject 5	Subject 6	Subject 7
Mean [s]	8.108	6.618	7.630	7.130	7.429	8.582	6.558
Q1 [s]	7.492	6.533	7.358	6.892	7.083	8.575	6.487
Q2 [s]	8.150	6.600	7.642	7.100	7.233	8.617	6.567
Q3 [s]	8.292	6.717	7.896	7.258	7.783	8.642	6.600

**Table 7:** Summary of the radius of curvature for Curve 1.

Subject	Subject 1	Subject 2	Subject 3	Subject 4	Subject 5	Subject 6	Subject 7
Mean [m]	9.207	8.121	5.886	8.069	8.570	8.555	8.127
Q1 [m]	8.136	7.536	5.597	7.262	7.660	7.200	8.011
Q2 [m]	9.303	8.060	5.973	7.802	7.974	8.039	8.232
Q3 [m]	9.946	8.747	6.238	8.892	9.407	9.653	8.490

**Table 8:** Summary of the radius of curvature for Curve 2.

Subject	Subject 1	Subject 2	Subject 3	Subject 4	Subject 5	Subject 6	Subject 7
Mean [m]	5.916	6.784	6.589	7.270	7.043	3.615	9.533
Q1 [m]	5.424	6.384	6.220	6.636	6.904	3.454	9.035
Q2 [m]	5.876	6.808	6.404	7.309	7.169	3.629	9.654
Q3 [m]	6.460	6.971	6.777	8.006	7.275	3.683	10.120

**Table 9:** Summary of the inter-camera junction discrepancy in the y-direction.

Subject	Subject 1	Subject 2	Subject 3	Subject 4	Subject 5	Subject 6	Subject 7
Mean [m]	0.083	0.082	0.042	0.04	0.124	0.031	0.042
Q1 [m]	0.035	0.057	0.026	0.027	0.072	0.012	0.026
Q2 [m]	0.085	0.089	0.043	0.041	0.116	0.025	0.040
Q3 [m]	0.128	0.112	0.051	0.047	0.166	0.041	0.055

## Data Availability

The data used in this work are available upon request to the corresponding author.
